# *Campylobacter jejuni* Cytolethal Distending Toxin Induces GSDME-Dependent Pyroptosis in Colonic Epithelial Cells

**DOI:** 10.3389/fcimb.2022.853204

**Published:** 2022-04-27

**Authors:** Jiayun Gu, Yan Lin, Zhichao Wang, Qicong Pan, Guohua Cai, Qigai He, Xiaojuan Xu, Xuwang Cai

**Affiliations:** ^1^State Key Laboratory of Agricultural Microbiology, College of Veterinary Medicine, Huazhong Agricultural University, Wuhan, China; ^2^Key Laboratory of Preventive Veterinary Medicine in Hubei Province, The Cooperative Innovation Center for Sustainable Pig Production, Wuhan, China

**Keywords:** *Campylobacter jejuni*, cytolethal distending toxin, pyroptosis, GSDME, cell death

## Abstract

**Background:**

Cytolethal distending toxin (CDT) is a critical virulence factor of *Campylobacter jejuni*, and it induces cell death and regulates inflammation response in human epithelial cells. Pyroptosis is an inflammatory form of programmed cell death (PCD), but whether it is involved in CDT-mediated cytotoxicity remains elusive.

**Aims:**

This study explores the role and mechanism of pyroptosis in CDT-mediated cytotoxicity.

**Methods:**

HCT116 and FHC cell lines were treated with CDT. Cell Counting Kit-8 (CCK-8) assay was used to detect cell viability. Western blotting was used to measure the expression of related proteins in the pathway, and cell morphology observation, annexin V/propidium iodide (PI) staining and lactate dehydrogenase (LDH) release assay were performed to evaluate the occurrence of pyroptosis.

**Result:**

Our results show that *C. jejuni* CDT effectively induces pyroptosis in a dose- and time- dependent manner in human colonic epithelial cells owing to its DNase activity. Specific pyroptotic features including large bubbles emerging from plasma membrane and LDH release were observed upon CDT treatment. Moreover, CDT-induced pyroptosis involves the caspase-9/caspase-3 axis, which is followed by gasdermin E (GSDME) cleavage rather than gasdermin D (GSDMD). N-acetyl cysteine (NAC), a reactive oxygen species (ROS) inhibitor, attenuates the activation of caspase-9/3, the cleavage of GSDME and pyroptotic characteristic, therefore demonstrating ROS initiates pyroptotic signaling.

**Conclusions:**

We first clarify a molecular mechanism that CDT induces pyroptosis *via* ROS/caspase-9/caspase-3/GSDME signaling. These findings provide a new insight on understanding of CDT-induced pathogenesis at the molecular level.

## Introduction

*Campylobacter jejuni* is the leading cause of bacterial foodborne gastroenteritis in the world, which is responsible for at least 96 million cases of enteric infection globally each year (Elmi et al., 2020). Several virulence factors are involved in the persistent infection and pathogenic process, of which cytolethal distending toxin (CDT) is the major bacterial cytotoxin that contributes to *C. jejuni*-related disease progression (Smith and Bayles, 2006). CDT has also been found to be expressed by more than 30 phylogenetically distant proteobacteria, including *Escherichia coli*, *Haemophilius ducreyi*, *Helicobacter hepaticus*, *Haemophilus parasuis* and *Aggregatibacter actinomycetemcomitans* (Pickett et al., 1996; Cope et al., 1997; Sugai et al., 1998; Ge et al., 2007; Zhang et al., 2012). However, very little is known about the role of CDT in the pathogenesis of *C. jejuni*. Our deficient understanding of CDT-host interactions remains a barrier to develop specific treatments.

CDT is a heterotrimeric holotoxin composed of three subunits, CdtA, CdtB and CdtC respectively, with CdtB being the most conserved one among CDT-harboring bacteria that exerts its effect as a DNase-I like property. CdtA and CdtC, served as regulatory subunits, are thought to attach to the cholesterol-rich lipid rafts on cell membranes, which enhance CdtB to enter into cells by endocytosis and translocate into nucleus eventually (Guerra et al., 2011; Bezine et al., 2014). Several lines of evidence suggest that the CDT-intoxicated cells undergo DNA double-strand breaks (DSB) followed by irreversible cell cycle arrest in G1 and/or G2 phase to allow DNA repair and cell survival, while cell death occurring if the damage beyond repair (He et al., 2021). As far as we know, CDT can trigger senescence, apoptosis and autophagy *in vitro* in a cell type-dependent manner and promote inflammation *in vivo* (Scuron et al., 2016; Li et al., 2017; Seiwert et al., 2017; Mathiasen et al., 2021). In epithelial cells, however, whether CDT induces other types of programmed cell death (PCD), such as pyroptosis, a type of pro-inflammation cell death, has not been reported.

Pyroptosis, a newly discovered form of PCD, is characterized by cell swelling with large bubbles emerging from plasma membrane and cell membrane rupture, resulting in the release of cellular contents eventually (Man et al., 2017). Originally, as a pro-inflammatory form of PCD, pyroptosis was known for mediation by pro-inflammatory caspases, including caspase-1 that often activated by canonical inflammasomes, and caspase-4/5/11 that often activated by non-canonical inflammasomes (Latz et al., 2013; Khan et al., 2018). But more recently, pyroptosis was also shown to be mediated by virulence factors, granzyme and pro-apoptotic caspases, suggesting pro-inflammatory caspases are not the unique inducer of pyroptosis (Wang et al., 2017; Zhou et al., 2020; Deng et al., 2022). Pyroptosis has been defined as a type of PCD relying on gasdermin protein family, which is the most notable feature distinguished from other PCDs (Shi et al., 2017). Emerging studies show that pyroptosis can be triggered by gasdermin D (GSDMD) or gasdermin E (GSDME), which was cleaved into N-terminal and C-terminal fragments by the active caspase-1/4/5/11 or caspase-3 respectively (Shi et al., 2015; Wang et al., 2017). The generating GSDMD-N or GSMDE-N fragment then oligomerizes and forms membrane pores, leading to cell swelling and perforation (Ding et al., 2016). Interestingly, differ to GSDMD-dependent pyroptosis, GSDME is able to switch caspase-3 mediated apoptosis to pyroptosis after pro-apoptotic stimulation treatment (Wang et al., 2017). As we know, CDT-mediated apoptosis has been reported to follow the mitochondrial pathway (Liyanage et al., 2010; Li et al., 2017). Together, these observations suggest a possible link between CDT and pyroptosis that requires elucidation.

In the present work, we illuminate that the presence of CjCDT induces DNA damage and inhibits cell viability in HCT116 and FHC cell lines. Additionally, our findings also show that GSDME-mediated pyroptosis is induced in response to CDT through the activation of reactive oxygen species (ROS)/caspase-9/caspase-3 pathway.

## Materials and Methods

### Antibodies and Reagents

Antibodies for GSDME (ab215191), GSDMD (ab209845) were purchased from Abcam (Cambridge, Cambridgeshire, Britain). Antibodies for caspase-1 (3866T), caspase-9 (9502T), caspase-3 (9668T), caspase-8 (9746T) and γH2AX (9718T) were purchased from Cell Signaling Technology (Danver, MA, USA), and GAPDH (60004-1-lg) was from Proteintech Group (Wuhan, Hubei, China). The secondary antibodies including HRP Goat Anti-Mouse IgG (H+L) (AS003), HRP Goat Anti-Rabbit IgG (H+L) (AS014) and FITC Goat Anti-Rabbit IgG (H+L) (AS011) were bought from Abclonal (Wuhan, Hubei, China).

Other reagents were purchased as follows: caspase-3 inhibitor (Z-DEVD-FMK) (HY-12466) and ROS scavenger N-acetyl cysteine (NAC) (HY-B0215) were from MedChemExpress (Monmouth Junction, NJ, USA); protease inhibitor cocktail tablets (BL612A) were from Biosharp (Beijing, China).

### Cell Culture and Treatment

Human colorectal cancer cell line HCT116 cells were obtained from Shanghai Institute of Cell Biology, Chinese Academy of Sciences. Human colonic epithelial cell line FHC cells were purchased from Kunming Cell Bank, Kunming Institute of Zoology. HCT116 and FHC cells were cultured in Dulbecco’s Modified Eagle Medium (DMEM)-high glucose supplemented with 10% fetal bovine serum (FBS) (Gibco, Grand Island, NY, USA) and 1% penicillin-streptomycin (Gibco, Grand Island, NY, USA). The cells were incubated at 37°C incubator containing 5% CO_2._

For CDT treatments, cells were pre-seeded in 12-well plates overnight until the cells reached 50% confluency. To inhibit the activity of caspase-3 or ROS, cells were pre-treated with Z-DEVD-FMK for 2 h or NAC for 3 h, respectively. Unless otherwise specified, the final action concentrations were: 10 μg/ml for CDT, 40 μM for Z-DEVD-FMK and 5 μM for NAC.

### Western Blotting

Both cells and supernatants were collected for Western blot. After PBS washing twice, the cell sediments were lysed with radio-immuno-precipitation assay (RIPA) buffer (Beyotime, Shanghai, China) with protease inhibitor cocktail and the protein concentrations were measured by Bicinchoninic Acid (BCA) Protein Assay Kit (Biosharp, Beijing, China) finally. Total 40 μg of protein from each sample was separated by SDS-PAGE and transferred to a PVDF membrane followed by blocking with 5% non-fat dry milk for 1 h at room temperature. The membrane was then incubated with primary antibodies at 4°C overnight, followed by incubated with HRP-conjugated secondary antibodies for 1 h at room temperature. The protein bands were identified using Clarity Max Enhanced Chemiluminescence (ECL) Western Blotting Substrates (Bio-Rad, Hercules, CA, USA).

### Cell Viability Assay and LDH Release Assay

For detecting cell viability, cells were pre-seeded into 96-well plates (1.2 × 10^4^ cells/well) and treated with CDT for 24 h. Cell viability was measured using Cell Counting Kit-8 (CCK-8) assay (Biosharp, Beijing, China) according to the manufacturer’s protocol.

The release rate of lactate dehydrogenase (LDH) was examined using LDH Cytotoxicity Assay Kit (Beyotime, Shanghai, China) according to the manufacturer’s protocol. The absorbance at 490 nm was detected after incubation. All experiments were repeated three times.

### ROS Measurement

The intracellular ROS levels were detected by ROS Assay Kit (Beyotime, Shanghai, China). After CDT exposure for 24 h, cells were incubated in the medium containing DCFH-DA (10 μM) at 37°C for 30 min in dark, followed by washing three times with non-FBS medium. The ROS levels were measured by a flow cytometer (BD Bioscience, Bedford, MA, USA).

### Cell Death Evaluation by Flow Cytometry

Cells were pre-seeded into 12-well plates (2.5 × 10^5^ cells/well) and treated with CDT for 24 h. CDT-induced cell death was detected with annexin V-fluorescein isothiocyanate (FITC) and propidium iodide (PI) Apoptosis Detection Kit (Yeasen, Shanghai, China). After treatment, cells were harvested, washed with PBS and incubated in annexin V and PI for 10 min at room temperature in dark. 1.0 × 10^4^ cells were collected and detected with flow cytometer (FACSVerse, Bioscience, CA, USA), and data was analyzed by FlowJo software. Before the analysis, the gating was performed to exclude doublets and debris. Most of cells should be gated, and greater than 90% of surviving cells in the control group should be ensured, while dividing four rectangular gates. All assays were repeated three times.

### Expression and Purification of CDT

Recombinant CdtA, CdtB and CdtC from *C. jejuni* were overexpressed respectively as a fusion protein with histidine-tag (His-tag) in *E. coli* BL21 (DE3). The *E. coli* BL21 (DE3) containing expression plasmid was cultured and induced by 0.8 mM isopropyl-β-D-thiogalactopyranoside (IPTG) at 18°C for 20 h until an OD_600_ of 0.5–0.6. The cells were lysed, and recombinant proteins were purified *via* Ni-NTA affinity chromatography (GE healthcare, Marlborough, MA, USA) followed by SDS-PAGE and Western blotting. Three subunits were reconstituted as a holotoxin at 25°C for 1 h for subsequent research.

### CRISPR-Cas9 Knockout and siRNA Knockdown

The *GSDME*-KO cell lines were generated using CRISPR-Cas9 technology. In brief, the guide RNA (gRNA), 5ʹ-TAAGTTACAGCTTCTAAGTC-3ʹ to target *GSDME* was subcloned into lentiCRISPR v2 vector. The lentiviruses were collected after co-transfection of HEK293T cells with lentiviral vector and packaging plasmids for 48 h and then used to infect HCT116 cells. The infected HCT116 cells were selected with puromycin (1 μg/ml) for 3 days and the single clones were cultured in 96-well plates for another 10–14 days or longer. Anti-GSDME immunoblotting and sequencing were used to identify *GSDME*-KO cell lines.

For siRNA knockdown, 80 pmol siRNA was transfected into HCT116 cells using jetPRIME^®^ transfection reagent (Polyplus, Paris, France) following the manufacturer’s instruction. Cells were used for assay 48 h after transfection.

### The Single Cell Gel Electrophoresis Assay (Comet Assay)

DNA damage in single cells was detected using comet assay according to the article (Lu et al., 2017). Briefly, after the formation of agarose coat on the slide, cell suspension at a concentration of 2.0 × 10^5^/ml was mixed with 1% low-melting agarose at a ratio1:10 (v/v), and the mixture was immediately pipetted onto the slide. Following a solidification at 4°C for 30 min and lysis in lysis solution at 4°C for 1 h, the slides were transferred to alkaline electrophoresis solution for 1 h to allow DNA unwinding. Then, alkaline electrophoresis was performed for 30 min at 26 V and 300 mA. Slides were washed twice in dH_2_O and one time in 70% EtOH for 5 min each at room temperature, dried at 37°C for 15 min, and stained with 100 μl SYBR Green for 15 min. Comet images were taken using a fluorescence microscope (Olympus, Tokyo, Japan) and the images of comet assay from the experiment with at least 50 cells each group was analyzed with OpenComet plug-in in ImageJ. The tail moments are calculated as follow: TM=Tail length × Tail% DNA/100.

### Microscopy Imaging

To examine the morphological characteristics of pyroptosis, cells were pre-seeded into 12-well plates overnight until reaching approximately 50% confluency. After CDT exposure, static bright field images of cells were captured using Ti-U microscope (Nikon, Tokyo, Japan).

To examine the γH2AX foci, cells were pre-seeded into 12-well plates overnight and subjected to different treatments. Afterward, the cells were incubated with 4% paraformaldehyde for 30 minutes, 0.5% Triton X-100 in PBS for 15 min, and blocked with 5% non-fat dry milk for 1 h at room temperature. The cells were then incubated with primary antibodies at 4°C overnight, followed by incubated with FITC-conjugated secondary antibodies for 1 h and DAPI for 15 min at room temperature. The fluorescence intensity was detected with the laser confocal microscope (Nikon, Tokyo, Japan).

### Statistical Analysis

Data were presented as mean ± standard error of the mean (SEM) from at least three experiments and were compared by one-way ANOVA followed by Tukey’s multiple comparison. Statistical analyses were performed using GraphPad Prism version 7.0 (CA, USA). *P < 0.05 was considered statistically significant, **P < 0.01 and ***P < 0.001 indicated extremely significant differences.

## Results

### The Cytotoxicity of CDT on Human Colonic Epithelial Cells

**Figure 1:**
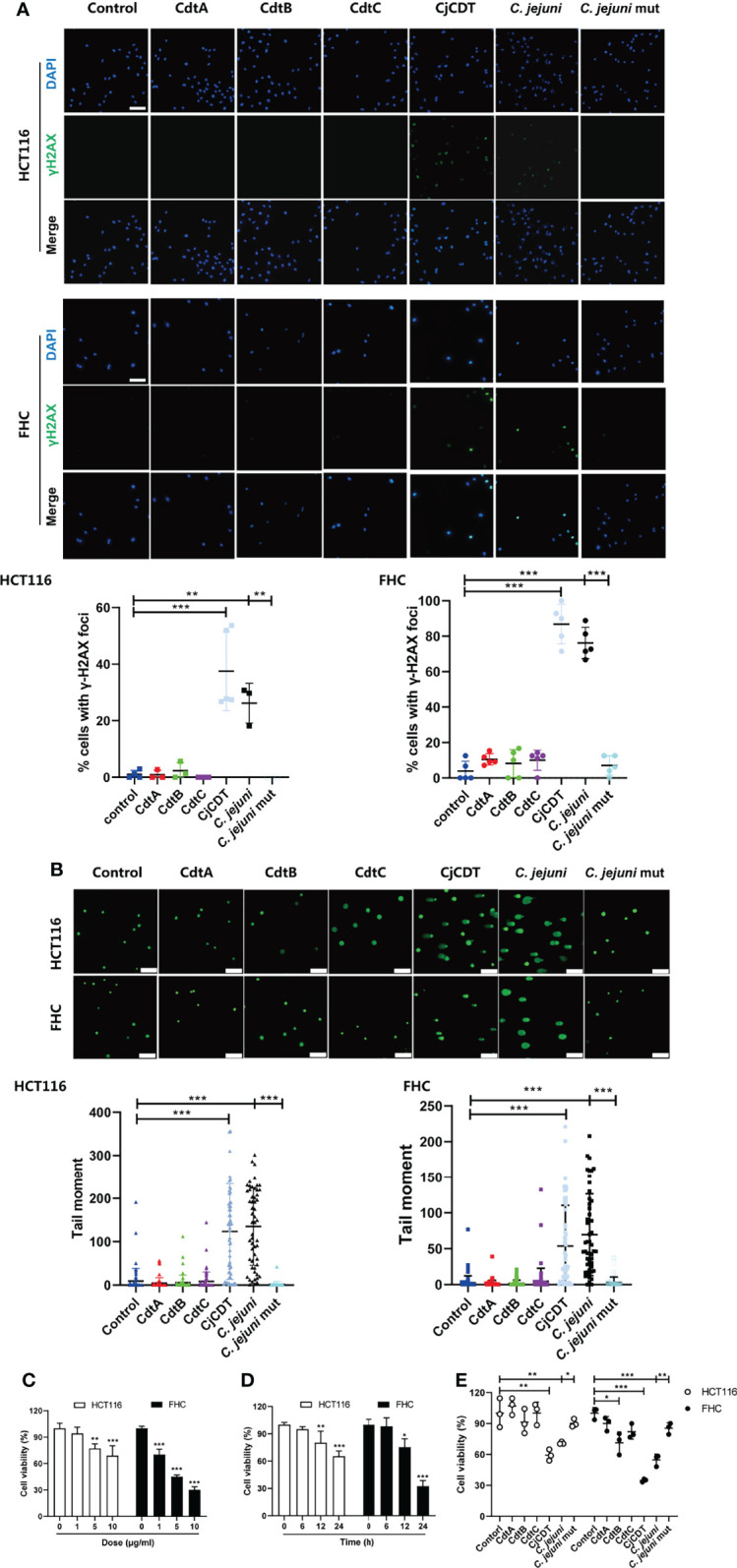
CjCDT induces DNA damage and inhibits cell viability in HCT116 and FHC cells. Cells were seeded into 12-well culture plates and exposed to CdtA, CdtB, CdtC, holotoxin CDT (CjCDT) for 24 h, and bacterial lysates from *C. jejuni* WT strain (*C. jejuni*) and *C. jejuni* mutant strain (*C. jejuni* mut) for 12 h. **(A)** Immunofluorescence microscopy was used to detect the DNA damage marker γH2AX. Scale bar 100 μm **(B)** Comet assay was performed to detect the DNA damage. Scale bar 200 μm **(C)** Cells were exposed to different concentrations of CjCDT (0, 1, 5, and 10 μg/ml) for 24 h. Cell viability was then detected using CCK-8 assay (n=3). **(D)** Cells were exposed to 10 μg/ml CjCDT for different periods of time (0, 6, 12 and 24 h). Cell viability was then detected using CCK-8 asssay (n=3). **(E)** Cells were exposed to 10 μg/ml CdtA, CdtB, CdtC or holotoxin CDT (CjCDT) for 24 h and bacterial lysates for 12 h. Cell viability was then detected using CCK-8 assay (n=3). *P < 0.05, **P < 0.01, ***P < 0.001. versus control using one way ANOVA.

To investigate the cytotoxicity of *C. jejuni* CDT on human colonic epithelial cells, a CDT-deficient mutant of *C. jejuni* strain NCTC11168 was engineered *via* electroporation of a gene targeting vector into the NCTC11168 wild type (WT) strain, in which the consecutive genes *cdtA, cdtB* and *cdtC*, were replaced by *kanR*. Later, we prepared bacterial lysates from *C. jejuni* WT (*C. jejuni*) and mutant strain (*C. jejuni* mut), and stimulated human colonic epithelial cell line FHC and human colon cancer cell line HCT116, with these extracts (10 μg/ml) for 12 hours or recombinant protein CdtA, CdtB, CdtC and holotoxin CDT (CjCDT) (10 μg/ml) for 24 hours. As shown in **Figure 1A**, CjCDT and extracts from *C. jejuni* WT strain treatments increased the number of γH2AX foci, a marker for DNA damage signaling, in both HCT116 and FHC cells, when compared with untreated cells. Conversely, extracts generated from *C. jejuni* mutant strain and the single subunits lacked a notable effect on augmenting γH2AX level (**Figure 1A**). Evaluation of DNA damage *via* comet assay revealed an increase in DNA damage in HCT116 and FHC cells exposed to CjCDT and extracts from *C. jejuni* WT strain when compared with cells exposed to *C. jejuni* mutant extracts and the single subunits (**Figure 1B**). Moreover, CCK-8 assay was performed to further evaluate the effect of CDT on cytotoxicity in human colonic epithelial cells. We found that exposure of cells to CjCDT attenuated the cell viability in a concentration- and time- dependent manner (**Figures 1C, D****)**. Interestingly, the cells primed with CdtA, CdtB or CdtC protein alone did not exhibit significant decline in cell viability unless with CDT holotoxin, indicating that the catalytic subunit CdtB requires the assistance of CdtA and CdtC to perform a cytotoxic function (**Figure 1E**). Collectively, these data strongly support the notion that CDT induces cytotoxicity.

### CjCDT Changes Cellular Morphology and Induces Cell Death in HCT116 and FHC Cells

**Figure 2:**
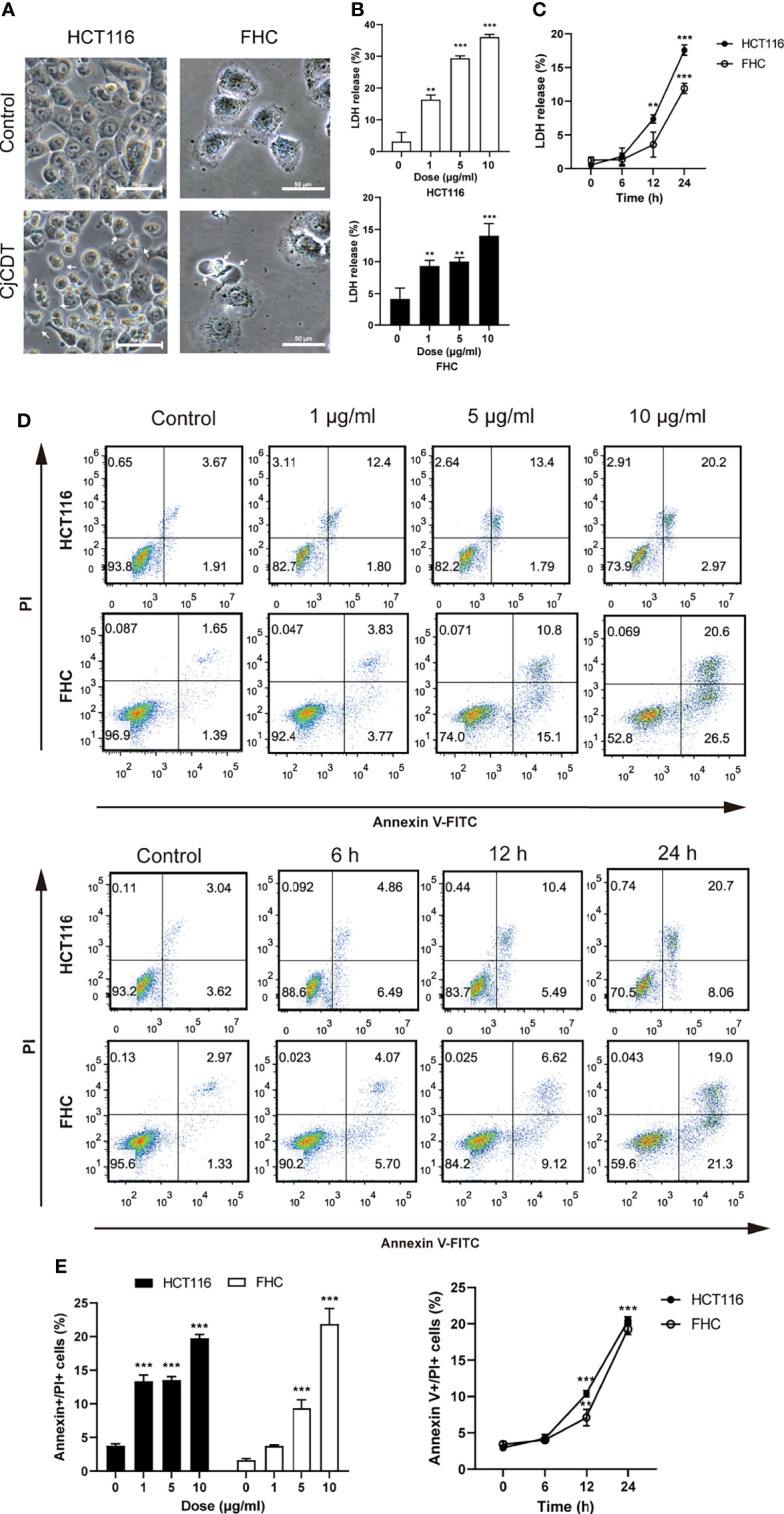
CjCDT changes cellular morphology and induces cell death in HCT116 and FHC cells. **(A)** After cells were treated with 10 μg/ml CjCDT for 24 h, representation microscopic images were taken. White arrowheads indicate the pyroptotic cells characterized with large bubbles emerging from plasma membrane. Scale bar 50 μm. **(B, C)** LDH release was measured after different doses (0, 1, 5 and 10 μg/ml) of CjCDT treatment for 24 h or 10 μg/ml CDT treatment for different periods of time (0, 6, 12 and 24 h) in HCT116 and FHC cells (n=3). **(D, E)** Annexin V-FITC/PI assay was performed to determine the pyroptotic cells after CjCDT treatment with different doses (0, 1, 5 and 10 μg/ml) for 24 h or 10 μg/ml CjCDT stimulation for different periods of time (0.6, 12 and 24 h), and analyzed by flow cytometry (n= 3). **P < 0.01, ***P < 0.001 versus control using one-way ANOVA.

Firstly, followed by the exposure of CDT, the morphological changes were observed. Morphologically, HCT116 and FHC cells exhibited microscopic features of cell swelling and balloon-like bubbles emerging from plasma membrane following CDT infection, which were different from the shrinkage of apoptotic cells (**Figure 2A**).The previous report found that pyroptosis is characterized by large bubbles blown from the plasma membrane, increase of LDH release and annexin V and PI positive cells (Rogers et al., 2017). It is plausible to postulate that pyroptosis is involved in CDT-mediated cytotoxicity. Afterward, HCT116 and FHC cells were stimulated with different concentrations (0, 1, 5 and 10 μg/ml) of CDT for 24 h or with a concentration of 10 μg/ml CDT for different periods of time (0, 6, 12 and 24 h). LDH release assay indicated that CDT increased LDH release in a dose- and time- dependent manner, suggesting the cell membrane rupture (**Figures 2B, C****)**. Moreover, flow cytometry analysis revealed that CDT treatment remarkably augmented the proportion of annexin V and PI double positive cells in a dose- and time- dependent manner, indicating the cell death through triggering plasma membrane permeabilization (**Figures 2D, E****)**. Hence, these results demonstrate that CDT changes cellular morphology and induces cell death in human colonic epithelial cells through triggering plasma membrane permeabilization.

### GSDME but Not GSDMD Is Involved in CDT-Triggered Pyroptosis in Human Colonic Epithelial Cells

**Figure 3:**
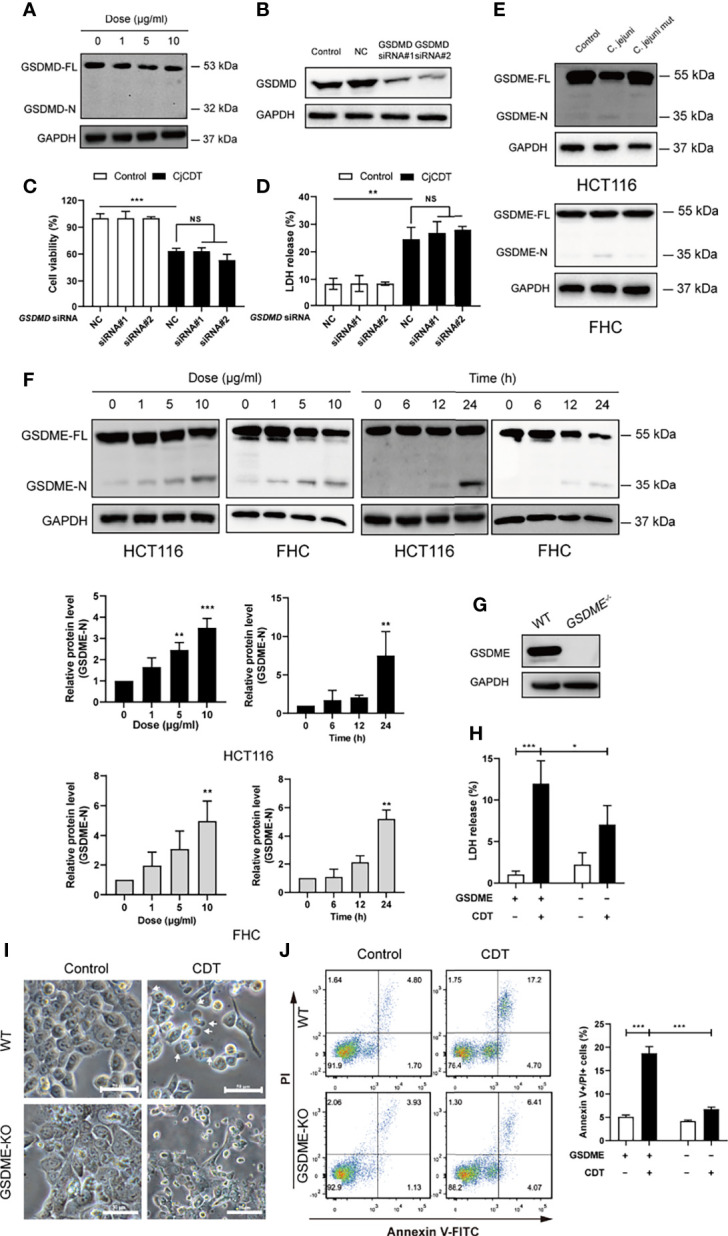
GSDME rather than GSDMD is essential for CjCDT induced-pyroptosis **(A)** GSDMD full length (GSDMD-FL) and N-terminus of GSDMD (GSDMD-N) were detected in HCT116 cells by Western blotting after CDT exposure (n=3). **(B)** The expression of GSDMD was detected in HCT116 cells by Western blotting after transfection of siRNA (n=3). **(C)** Cell viability was detected using CCK-8 assay after the transfected HCT116 cells were exposed to 10 μg/ml CjCDT for 24 h (n=3). **(D)** LDH release was then measured after the transfected HCT116 cells were exposed to 10 μg/ml CjCDT for 24 h. **(E)** GSDME full length (GSDME-FL) and N-terminus of GSDME (GSDME-N) were detected by Western blotting after bacterial lysates exposure (n=3) **(F)** GSDME full length (GSDME-FL) and N-terminus of GSDME (GSDME-N) were detected by Western blotting after CjCDT exposure (n=3) **(G)** GSDME knockout cell line was identified using Western blotting. **(H)** LDH release assay (n=3), **(I)** microscopic imaging and **(J)** annexin V-FITC/PI assay (n=3) were used to explore the role of GSDME in pyroptosis (n=3). White arrowheads indicate the pyroptotic cells characterized with large bubbles emerging from plasma membrane Scale bar 50 μm. NS, no significant difference, *P < 0.05, **P < 0.01, ***P < 0.001 versus control using one-way ANOVA.

Next, we moved on to explore whether pyroptosis is involved in CDT-mediated cytotoxicity and the mechanism by which CDT induces pyroptosis. First, to investigate whether GSDMD is involved in CDT-induced cytotoxicity, full-length GSDMD and cleaved N-terminus of GSDMD were detected using immunoblotting. We found that although GSDMD was expressed in HCT116 cells, no GSDMD cleavage was observed upon CDT treatment (**Figure 3A**). To further determine whether GSDMD participates in CDT-induced cytotoxicity, GSDMD was stably knocked down in HCT116 cells by transfecting siRNA targeting *GSDMD* (**Figure 3B**). Cell viability and LDH release assay revealed no obvious difference among the groups transfected with control siRNA or *GSDMD* targeting siRNA followed by CDT treatment, demonstrating that GSDMD is not involved in CDT-induced pyroptosis in HCT116 cells (**Figures 3C, D****)**. Given that GSDME is a newly recognized executor of cell pyroptosis, we postulated that GSDME can trigger CDT-induced pyroptosis. As expected, we found that CDT treatment (CjCDT and bacterial lysates from *C. jejuni* WT strain) led to elevated levels of N-terminus of GSDME in a dose- and time- dependent manner in HCT116 and FHC cells (**Figures 3E, F****)**. To further confirm this hypothesis, we knocked out the *GSDME* gene in HCT116 cells (**Figure 3G**). Knockout of GSDME resulted in significantly lessened LDH release and microscopic feature of large bubbles (**Figures 3H, I****)**, compared with negative control (NC) cells in response to CDT. Flow cytometry analysis revealed that GSDME knockout noticeably decreased the percentage of annexin V and PI double positive of HCT116 cells following CDT treatment (**Figure 3J**). These results suggest that lack of GSDME diminished CDT-induced pyroptosis. In conclusion, these results demonstrate that GSDME is a key executive protein in CDT-mediated pyroptosis.

### Activation of Caspase-9/Caspase-3 Is Associated With GSDME Cleavage in CDT-Induced Pyroptosis

**Figure 4:**
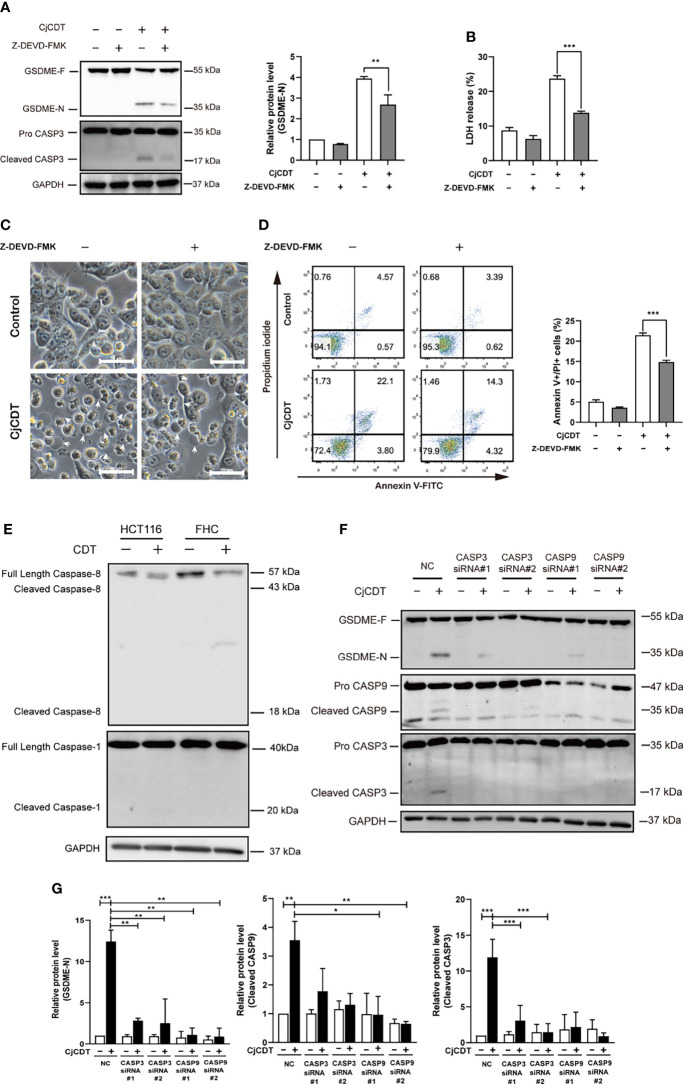
Activation of caspase-9/caspase-3 contributes to CjCDT induced-pyroptosis. HCT116 cells were exposed to CjCDT (10 μg/ml) in the absence or presence of caspase-3 inhibitor Z-DEVD-FMK, and incubated for 24 h. **(A)** The expression of pyroptosis executive protein GSDME and cleaved caspase-3 was detected using Western blotting (n=3). **(B)** Release of LDH was detected in the culture supernatants (n=3) **(C)** Representation microscopic images were taken. White arrowheads indicate the pyroptotic cells characterized with large bubbles emerging from plasma membrane Scale bar 50 μm. **(D)** Cell death was evaluated by staining with annexin V-FITC and PI, and analyzed by flow cytometry (n=3). **(E)** The activation of caspase-8 and caspase-1 was detected using Western blotting (n=3). **(F, G)** After cells were transfected with caspase-3, caspase-9 or negative control siRNA followed by CjCDT stimulation, the expression of GSDME, cleaved caspase-3 and caspase-9 was detected using Western blotting (n=3). *P < 0.05, **P < 0.01, ***P < 0.001 versus control using one-way ANOVA.

We next sought to explore the potential molecular mechanism of GSDME-mediated pyroptosis induced by CDT. To this end, firstly, HCT116 cells were exposed to CDT in the absence or presence of the caspase-3 specific inhibitor, Z-DEVD-FMK, and incubated for 24 h. A notable attenuation was observed in terms of GSDME cleavage (**Figure 4A**), LDH release (**Figure 4B**), microscopic feature of large bubbles (**Figure 4C**) and cell death (**Figure 4D**) in combined treatment group, compared with the cells stimulated with CDT separately, demonstrating the role of caspase-3 in the process. It is well known that caspase-3 is an executor caspase that can be activated either by caspase-9 *via* mitochondrial pathway or caspase-8 *via* death receptor pathway (Chen et al., 2006). In the present study, we revealed that caspase-8 was not activated after HCT116 and FHC cells were exposed to CjCDT (**Figure 4E**). Besides, no evident activation of pro-inflammatory caspase, caspase-1, was found as well, indicating that CDT might not lead to pro-inflammatory effect in HCT116 and FHC cells directly (**Figure 4E**). Next, the siRNA technology was employed to knock down the expression of caspase-3 and caspase-9 for further investigation. Knocking down either caspase-3 or caspase-9 efficiently suppressed the cleavage of GSDME (**Table 1**). Additionally, caspase-9 knock-down severely compromised the activation of caspase-3, whereas caspase-3 knock-down lacked a notable inhibition on caspase-9 activation (**Figures 4F, G****)**. Together, these results demonstrate that caspase-9/caspase-3/GSDME axis is associated with CDT-induced pyroptosis.

**Table 1:** Related siRNA sequences used in the experiment.

Gene	Forward	Reverse
GSDMD#1	GGAGACCAUCUCCAAGGAA	UUCCUUGGAGAUGGUCUCC
GSDMD#2	GGAACUCGCUAUCCCUGUU	GGAACUCGCUAUCCCUGUU
Caspase-3#1	GGAACCAAAGAUCAUACAU	AUGUAUGAUCUUUGGUUCC
Caspase-3#2	GCCGACUUCUUGUAUGCAU	AUGCAUACAAGAAGUCGGC
Caspase-9#1	GCAAAGUUGUCGAAGCCAA	UUGGCUUCGACAACUUUGC
Caspase-9#2	GCAGAAAGACCAUGGGUUU	AAACCCAUGGUCUUUCUGC
Negative control	UUCUCCGAACGUGUCACGU	ACGUGACACGUUCGGAGAA

Sequences: 5ʹ to 3ʹ.

### ROS Is Involved in CDT-Induced Pyroptosis

**Figure 5:**
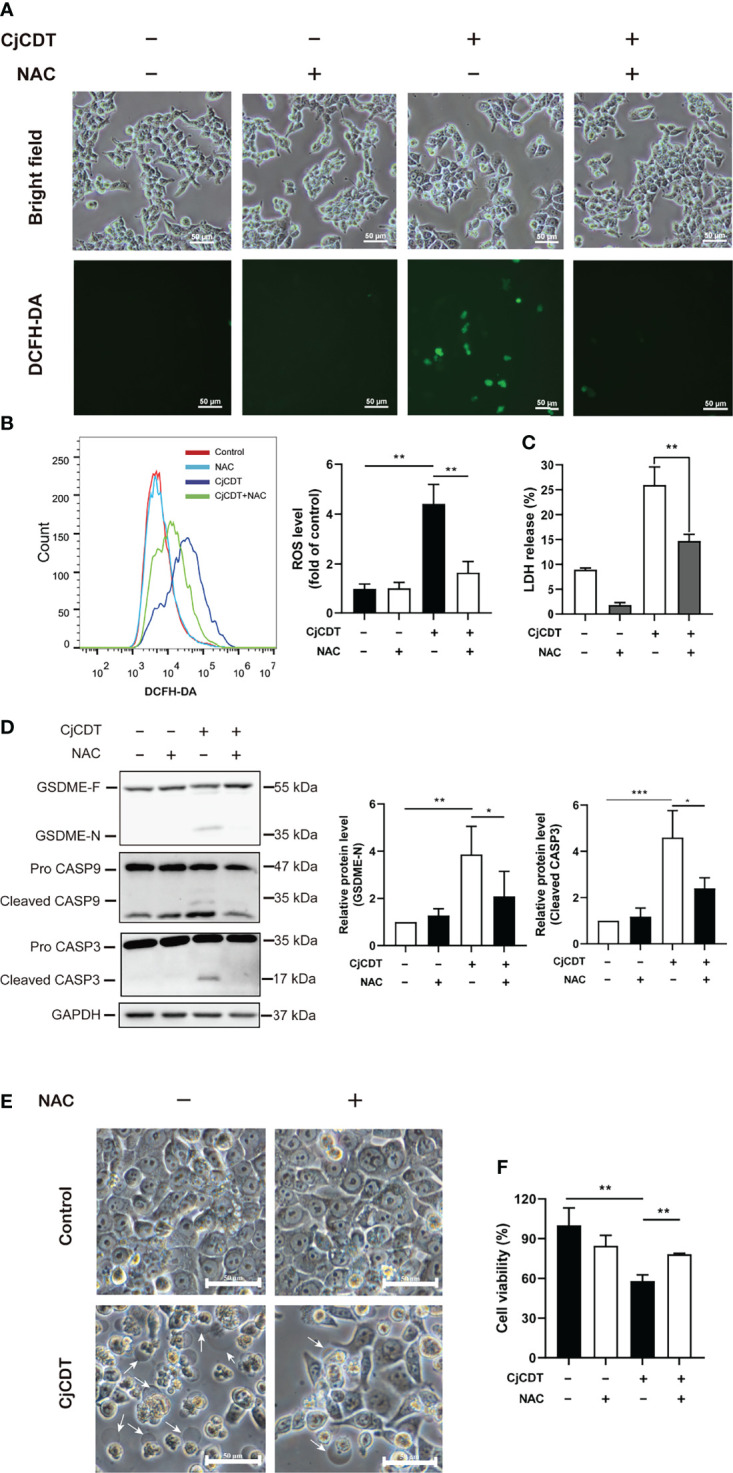
ROS participates in CjCDT induced-pyroptosis. HCT116 cells were exposed to CjCDT (10 μg/ml) in the absence or presence of ROS scavenger NAC, and incubated for 24 h. **(A)** ROS levels were determined by confocal microscopy. Scale bar 50 μm. **(B)** ROS levels were determined by flow cytometry (n=3). **(C)** Release of LDH was measured in the culture supernatant (n=3). **(D)** The expression of GSDME cleaved caspase-3 and caspase-9 was detected using Western blotting in (n=3). **(E)** Representation morphological alterations were taken. White arrowheads indicate the pyroptotic cells characterized by large bubbles emerging from plasma membrane. Scale bar 50 μm. **(F)** Cell viability was detected using CCK-8 assay, *P < 0.05, **P < 0.01, ***P < 0.001 versus control using one-way ANOVA.

Excessive accumulation of ROS could increase cellular oxidative stress, leading to several types of cell death (Zhou et al., 2018). However, whether ROS is related to CDT-induced pyroptosis remains currently unclear. First, we performed flow cytometry to detect the level of intracellular ROS following CDT treatment. Indeed, we found that CDT exposure significantly increased cellular ROS levels reflected by the fluorescence of DCFH-DA. Of note, the effect was impeded by NAC, a scavenger of ROS (**Figures 5A, B****)**. Then, we asked whether cellular ROS may influence CDT-induced cleavage of caspase-9/caspase-3/GSDME in HCT116 cells. As expected, the cells pretreated with NAC followed by CDT stimulation exhibited the attenuation of LDH release (**Figure 5C**), GSDME cleavage, caspase-3 and caspase-9 activation (**Figure 5D**). Moreover, CDT-induced pyroptotic morphology and cell death were rescued by NAC incubation (**Figures 5E, F****)**. These results indicate that the removal of ROS efficiently suppressed CDT-triggered pyroptosis. Hence, our results suggest that ROS, serving as an upstream of caspase-9/caspase-3/GSDME pathway, plays an essential role in CDT-mediated pyroptosis.

## Discussion

The present study was set out to explore the effect of *C. jejuni* CDT on human colonic epithelial cells and the role of pyroptosis in this process. Three major findings of our work are: (i) *C. jejuni* CDT could induce DNA damage, inhibit cell viability, and trigger cell death eventually, suggesting the damage of this pathogen to the integrity of intestinal tract epithelium; (ii) *C. jejuni* CDT could induce pyroptosis of colonic epithelial cells, which has never before been reported; (iii) Pyroptosis is induced through the ROS/caspase-9/caspase-3/GSDME pathway in response to *C. jejuni* CDT.

CDT is a crucial and special virulence factor in the pathogenic process of *C. jejuni*, and it is the only genotoxic toxin which expresses in almost all *C. jejuni* serotypes (Smith and Bayles, 2006). However, very little is known about the role of CDT in the pathogenesis of *C. jejuni*. In the present study, we found that both recombinant CDT and bacterial lysates from *C. jejuni* WT strain exhibited DNase activity, while *C. jejuni* mutant did not. Moreover, the CDT holotoxin rather than its individual subunits inhibited cell viability, indicating that CDT treatment leads to cell death ultimately.

Next, the molecular mechanism of CDT-induced cell death was addressed. The cytotoxicity of CDT has been previously bound up with endogenous apoptosis (Liyanage et al., 2010; Li et al., 2017). It is worth noting that with further research on other forms of PCD, however, remarkable flexibility and considerable degree of plasticity of their molecular regulation was revealed between different types of PCD (Bedoui et al., 2020). Pyroptosis is critical for host defenses against extracellular pathogens infection and danger signals due to the release of cytosolic contents that are rich in damage associated molecular patterns (DAMPs), which could activate other immune cells (Qu et al., 2020). At least four different pathways have been confirmed to initiate pyroptosis according to the executive gasdermin protein. The first is pathogen-activated pyroptosis pathway, in which GSDMD was cleaved by the activated caspase-1/4/5/11, resulting in the pore formation and membrane rupture (Shi et al., 2015). GSDMD-mediated pyroptosis is mainly stimulated by the activation of caspase-1 (canonical pathway) (Lamkanfi and Dixit, 2014) or caspase-4/5/11 (non-canonical pathway) (Shi et al., 2014). In canonical pathway, inflammasomes such as NLRP3, NLRP4 and AIM2 are responsible for recognizing extracellular pathogens infection and then activate the caspase-1, which cleaves GSDMD into the N-terminus and C-terminus fragments later. In non-canonical pathway, bacterial lipopolysaccharides (LPS) entering into the cytoplasm is sensed by its intracellular receptors, caspase-4/5 (human) and caspase-11 (mouse), leading to their activation followed by the cleavage of GSDMD (Shi et al., 2014). Granzyme A-directed cleavage of gasdermin B (GSDMB) and caspase-8-directed cleavage of gasdermin C (GSDMC) have been proved to trigger pyroptosis as well (Hou et al., 2020; Zhou et al., 2020). The fourth is induced by certain apoptotic stimuli, where activated caspase-3 cleaves GSDME, switching apoptosis to pyroptosis (Rogers et al., 2017). In the present study, we first found that CDT induces pyroptosis in human colonic epithelial cells. We observed the characteristics of pyroptosis, including plasma membrane swelling, LDH release and augment of annexin V and PI positive cells, which differ from a number of studies showing that CDT triggers apoptosis *via* the mitochondrial pathway (Liyanage et al., 2010; Chen et al., 2017; Li et al., 2017). On the one hand, it cannot be ruled out that pyroptosis induced by CDT has been mistakenly thought as apoptosis in the past decade, because pyroptosis was not recorded as be a gasdermin-dependent PCD until 2015 (Shi et al., 2015). On the other hand, it is possible that apoptosis and pyroptosis were induced simultaneously, while apoptosis became the subject of research for the reason of inadequate understanding of pyroptosis in the past. Additionally, we found GSDME instead of GSDMD was cleaved, indicating that CDT probably triggers GSDME-mediated pyroptosis. Supporting this observation, knock-out of *GSDME* in HCT116 cells attenuated CDT-induced pyroptotic feature. *GSDME* knockout resulted in reduction of LDH release, large bubbles emerging from plasma membrane, and the percentage of annexin V/PI double positive cells, confirming the deceleration of CDT-induced pyroptosis. Interestingly, Shenker et al. revealed that *A. actinomycetemcomitans* CDT-treated macrophages exhibited GSDMD-mediated pyroptosis for its phosphatidylinositol-3,4,5-trisphosphate (PIP_3_) phosphatase activity (Shenker et al., 2020). Recently, PIP_3_ phosphatase activity of *C. jejuni* CDT was discovered (Huang et al., 2021). In the present study, however, no relationship was found between GSDMD and *C. jejuni* CDT-induced pyroptosis. The discrepancy between the results of two studies is likely that the DNase activity of CDT was more likely to be carried out in proliferating cells, whereas GSDMD-dependent pytoptosis triggered by PIP_3_ phosphatase activity of CDT occurred mainly in immune cells (Gargi et al., 2013; Liu et al., 2017).

Apoptosis and GSDME-dependent pyroptosis share some common components and features, among which caspase-3 is considered as the final executive protein that determines the type of cell death according to the cleaved substrate (Wang et al., 2017). During the process of CDT induced-pyroptosis, we observed the activation of caspase-3 in response to CDT. It is well known that caspase-3 is activated both by caspase-9 and caspase-8 *via* mitochondrial pathway and death receptor pathway, respectively (Chen et al., 2006). Death receptor pathway (extrinsic apoptosis pathway) is activated by some ligands of death receptor, such as TNF-α or FasL (Wang et al., 2012). But in the present study, there was no detectable activated caspase-8. We ruled out the possibility that caspase-8 is involved in the process of CDT-induced pyroptosis. In our case, we noticed the activation of caspase-9 after CDT treatment and the diminished cleavage of GSDME through *CASPASE-3* and *CASPASE-9* knock-down.

During the process of our experiment, *CASPASE-3* knock-down showed inadequate efficiency (40-50%) compared with *CASPASE-9*. Nevertheless, it still contributed to the inhibition of the cleavage of GSDME markedly. Thus, it confirms that CDT activates the intrinsic apoptosis pathway at first, in which activated caspase-3 cleaves GSDME, switching apoptosis to pyroptosis.

The production of intracellular ROS is correlated with DNA damage and this elevation in ROS levels is believed to play an essential role in regulating cell death and survival (Rowe et al., 2008; Hamanaka and Chandel, 2010). Several types of programmed cell death are related to the excessive accumulation of intracellular ROS, including apoptosis (Adachi et al., 2015), pyroptosis (Yang et al., 2020), autophagy (Li et al., 2015) and necrosis (Yang et al., 2018). Then, it is plausible to postulate that ROS initiates CDT-induced pyroptosis in human colonic epithelial cells. Indeed, our data demonstrate that the level of cellular ROS was significantly augmented after CDT exposure, and decreased while priming with the antioxidant, NAC, which impeded the cleavage of caspase-9, caspase-3 and GSDME, and pyroptosis consequently. Another study also demonstrated that iron-elevated ROS triggers pyroptosis by increasing the oxidation and oligomerization of Tom20, which promotes the Bax/caspase-9/caspase-3/GSDME pathway (Zhou et al., 2018). Besides, the accumulation of ROS caused by miltirone regulates the MEK/ERK1/2 signaling, which elicits Bax/caspase/GSDME pathway and triggers pyroptosis ultimately (Zhang et al., 2020). Based on these results, we conclude that ROS is involved in CDT-induced pyroptosis. But in our experiment, direct results suggesting the DNA damage is responsible for ROS production are insufficient, which is the limitation in this research as well. Further studies are needed to understand the relationship between DNA damage and ROS production and how ROS regulates the caspase-9/caspase-3/GSDME pathway after CDT treatment.

## Conclusions

**Figure 6:**
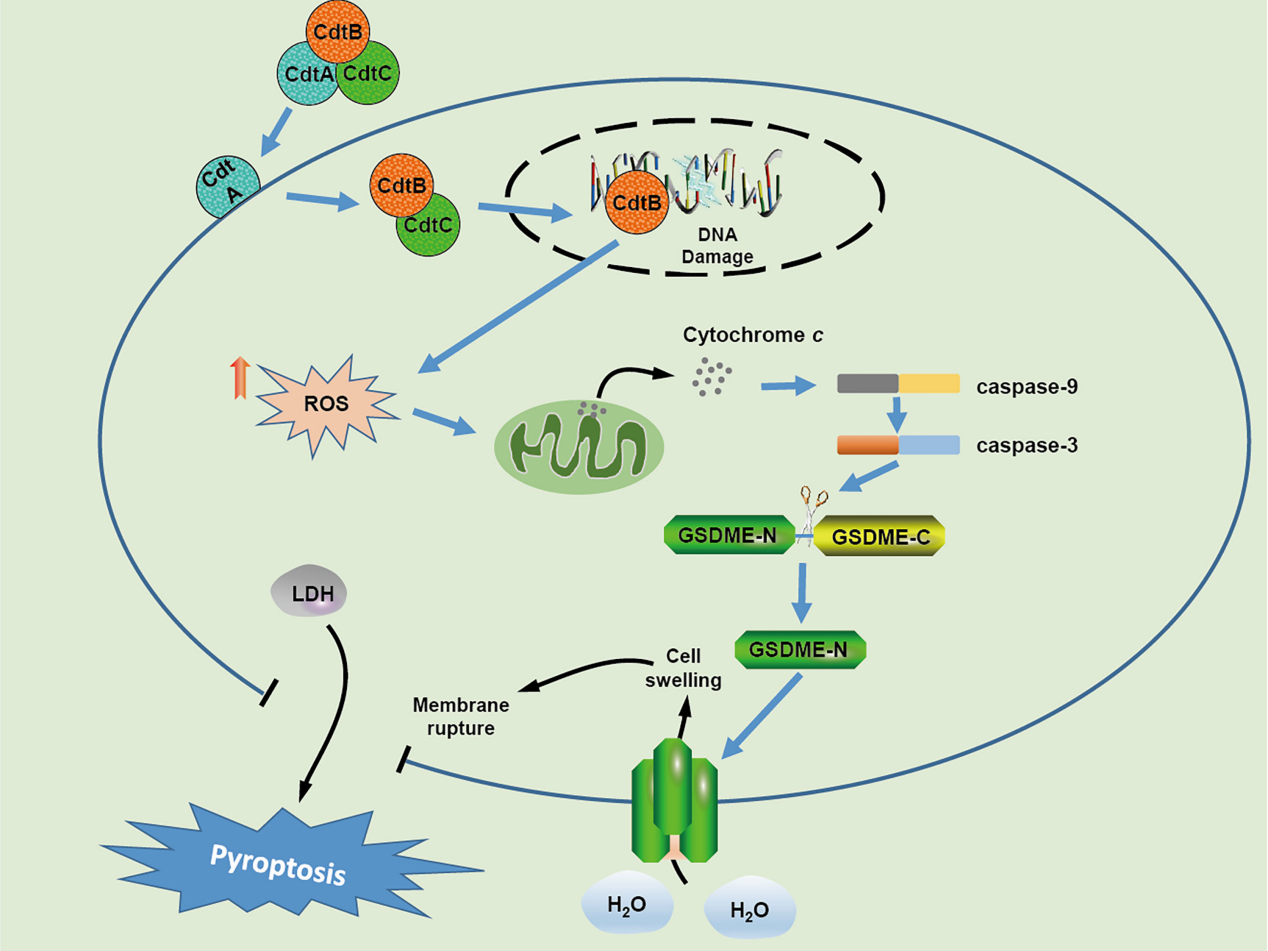
Schematic model of cellular mechanism underlying CDT induced pyroptosis in human colonic epithelial cells.In CDT treated-target cells, CdtB is relocated to the nucleus through the assistance of CdtA and CdtC, which results in DNA double strand breaks owing to its DNase activity. Upon DSB formation, the production of cellular ROS is increased and subsequently triggers the release of cytochrome c, followed by the activation of caspase-9 and caspase-3 The activated caspase-3 further cleaves GSDME, and eventually induces cell swelling and membrane rupture.

In conclusion, our results demonstrate that *C. jejuni* CDT triggers DSBs and inhibits cell viability in human colonic epithelial cells. Furthermore, CDT induces pyroptosis of human colonic epithelial cells *via* ROS/caspase-9/caspase-3/GSDME pathway (**Figure 6**). Determining the pathogenic mechanism of CDT may result in the development of new strategies for controlling bacterial infections.

## Data Availability Statement

The raw data supporting the conclusions of this article will be made available by the authors, without undue reservation.

## Author Contributions

JG contributed to study conceptualization, perform the experiment and edit the manuscript. YL performed the experiments. ZW, QP, and GC analyzed the data and contributed reagents, materials, and analysis tools. QH, XX, XC acquired the funding and provided overall supervision. All authors contributed to the article and approved the submitted version.

## Funding

This work was supported by the China Agriculture Research System of MOF and MARA.

## Conflict of Interest

The authors declare that the research was conducted in the absence of any commercial or financial relationships that could be construed as a potential conflict of interest.

## Publisher’s Note

All claims expressed in this article are solely those of the authors and do not necessarily represent those of their affiliated organizations, or those of the publisher, the editors and the reviewers. Any product that may be evaluated in this article, or claim that may be made by its manufacturer, is not guaranteed or endorsed by the publisher.
